# Denser forests across the USA experience more damage from insects and pathogens

**DOI:** 10.1038/s41598-023-30675-z

**Published:** 2023-03-04

**Authors:** Christopher Asaro, Frank H. Koch, Kevin M. Potter

**Affiliations:** 1 USDA Forest Service, State and Private Forestry, Forest Health Protection, Atlanta, GA 30309 USA; 2 Department of Forestry and Environmental Resources, North Carolina State University, Research Triangle Park, NC 27709 USA; 3 Present Address: USDA Forest Service, Southern Research Station, Research Triangle Park, NC 27709 USA

**Keywords:** Forest ecology, Forestry, Forest ecology, Forestry

## Abstract

Forests across much of the United States are becoming denser. Trees growing in denser stands experience more competition for essential resources, which can make them more vulnerable to disturbances. Forest density can be expressed in terms of basal area, a metric that has been used to assess vulnerability of some forests to damage by certain insects or pathogens. A raster map of total tree basal area (TBA) for the conterminous United States was compared with annual (2000–2019) survey maps of forest damage due to insects and pathogens. Across each of four regions, median TBA was significantly higher within forest areas defoliated or killed by insects or pathogens than in areas without recorded damage. Therefore, TBA may serve as a regional-scale indicator of forest health and a first filter for identifying areas that merit finer-scale analysis of forest conditions.

## Introduction

Reliable indicators of forest health are increasingly important under a changing climate where the scope and intensity of disturbances such as storms, wildland fire or insect and pathogen outbreaks may be more challenging to forecast^1–3^. Forest health is a difficult concept to define using terms such as integrity, resilience, or balance, which are problematic because they do not provide objective, scale-independent criteria that can easily be assessed quantitatively and applied consistently across forest ecosystems^1,4^. Contributing to the difficulty in defining forest health is the fact that the concept can encompass several dimensions, including tree damage and mortality, ecosystem function, sustainable use, and resistance to disturbance^3,5,6^. Additionally, definitions of forest health can be informed by either ecologically focused or utilitarian perspectives that may conflict^1,6^. Although tree mortality is not the only meaningful aspect of forest health, it is amenable to relatively straightforward measurement revolving around the baseline mortality of dominant tree species; that is, the degree to which actual mortality of these species across various age classes exceeds the average expected baseline mortality for each age class^1,7^. In other words, to evaluate this forest health component, we must be able to distinguish mortality of forest trees that surpasses natural (i.e. age- and density-dependent) mortality caused by successional processes or stand maturation^8,9^. Given the scope of challenges to forest ecosystems across the globe, there is a need for simple indicator variables that can be applied to forests at multiple scales so that potential for mortality above natural baseline levels–i.e. density-independent mortality^8^–can be identified. Ideally, any such indicator would also be applicable for forest damage other than mortality (e.g. defoliation above baseline levels).

One possible indicator variable, for instance, is the expression of forest density in terms of basal area, which is the sum of the cross-sectional areas of the trees in a locale of interest (e.g. all live trees in a forest stand) measured at breast height (1.37 m or 4.5 ft above ground) and expressed per unit of land area (m^2^ ha^−1^ or ft^2^ ac^−1^)^10^. As an indicator of forest density, basal area accounts for both the number of trees and the diameter distribution (i.e. the sizes) of those trees. While forest density can be defined more simply as the number of trees per unit area, basal area provides a fuller accounting of vegetative biomass and forest structure^11^. Basal area can also be expressed for individual tree species, genera, or any desired species grouping of interest. In this regard, it has been used as a key indicator of the risk of mortality from several forest insects associated with specific host trees^12,13^. However, to our knowledge, total basal area (i.e. the basal area of all live trees within a defined space) has never been evaluated quantitatively as an indicator of forest health on a regional or continental scale. Nor has total basal area been compared directly with spatiotemporal data on forest insect and pathogen disturbances across taxa, a relationship we explore herein for the conterminous United States (USA, lower 48 states). In this analysis, we tested the spatial association between total basal area across the USA and 20 years (2000–2019) of Insect and Disease Survey data collected and compiled by the Forest Health Monitoring Program of the USDA Forest Service in cooperation with state forestry agencies.

### ‘Host’ basal area versus ‘total’ basal area

The concept of higher basal area leading to greater risk of insect and pathogen disturbances is not new to forest ecologists. In the eastern USA, positive associations between high host basal area and disturbances have been demonstrated for the native southern pine beetle (*Dendroctonus frontalis*) and pines (*Pinus* spp.)^14^ as well as for the non-native spongy moth (*Lymantria dispar dispar*) and oaks (*Quercus* spp.)^13,15^. Across the western USA, several native bark beetles in the genus *Dendroctonus*, such as the mountain pine beetle (*D. ponderosae*), spruce beetle (*D. rufipennis*), and Douglas-fir beetle (*D. pseudotsugae*), are known to exploit high basal area stands of their associated coniferous hosts^12,16–18^. Another relevant example is the sirex woodwasp (*Sirex noctilio*), a pest of pines native to Europe that has invaded the northeastern USA and other parts of the world. A recent study^19^ reported strong relationships between basal area and sirex woodwasp mortality among pine stands on four continents, including sites in its native and invaded ranges.

Typically, these relationships are understood in the context of *host* basal area, where the hosts associated with an insect or pathogen may consist of one tree species or genus, several tree genera within a single plant family, or genera across multiple families for highly polyphagous species. For example, the spongy moth has a host range of hundreds of tree and shrub species in North America, although its strong preference for oaks leads to higher levels of defoliation and subsequent mortality in oak-dominated forests^13,15^. While it is logical at local scales to associate the risk and severity of a particular insect or pathogen with the basal area of its susceptible host trees, we propose that *total* basal area, irrespective of the host status of the trees comprising that total, is a useful predictor of conditions conducive to disturbance in general. The total basal area of a forest stand is one indicator of its vulnerability to tree stress due to competition; in short, a reduction in total basal area decreases competition among the stand’s trees for key resources (e.g. water, nutrients, light), decreasing stress and, by extension, the rate of tree mortality^20^. This concept has been explored in the context of facilitating forest adaptation to increased drought under a warming climate^20,21^. We assert, as have others^22,23^, that it has similar implications for forest insect and pathogen disturbances.

Viewing total basal area in relation to cumulative insect and pathogen disturbances across taxa instead of host basal area in relation to specific disturbances can help explain several observations that may, at first, seem counterintuitive. For example, in the USA, federal lands such as national parks or national forests, which tend to be more biodiverse and contain more contiguous and mature forest, may experience more frequent or severe disturbances from insects and pathogens than surrounding forested landscapes that are mostly privately owned, more fragmented, less biodiverse, more intensively managed, and where trees are younger on average^15,24,25^. Although structural and tree species diversity tends to make forests resistant to such disturbances^26–28^, at least those caused by native insects or pathogens, this “associational” resistance may be counteracted by high basal area conditions^29^. For instance, in an analysis of mountain pine beetle impacts in the Black Hills region of South Dakota, mortality risk increased similarly with higher non-host basal area as it did with higher host basal area^30^. In other words, trees in homogeneous stands were as likely to die as trees in comparatively heterogeneous stands, possibly due to competition for moisture among all trees, both hosts and non-hosts^30^. There are other examples of total basal area as an informative metric separate from host basal area. In an analysis of a drought-associated outbreak of fir engraver (*Scolytus ventralis*) in the Sierra Nevada Mountains of California, the model that best predicted white fir (*Abies concolor*) mortality included both white fir (i.e. host) basal area and total basal area as predictors^31^. During the last 15 years, southern pine beetle outbreaks have been observed more frequently in some mountainous areas of the southeastern USA dominated by hardwoods with a pine component (e.g. montane oak-hickory or oak-pine forests) than in areas where pure pine plantations are far more abundant and widespread; stress on the host pines from competition, especially with the hardwoods, may have been a contributing factor^32,33^. Similarly, the probability of eastern larch (*Larix laricina*) mortality during an outbreak of eastern larch beetle (*Dendroctonus simplex*) in Minnesota was positively related to non-host conifer basal area and tree diameter. As in the preceding case, stress due to competition from the non-hosts was suggested as a possible explanation^34^.

These examples can be adequately explained by forest ecologists based on the biology of the insect or pathogen in question, the prevalence of its hosts, and key forest characteristics (e.g. site quality, age structure, species composition). Nevertheless, a commonality among the examples is that higher total basal area is associated in some regard with a greater risk of insect or pathogen disturbance. Of course, such disturbances are not universally detrimental to forest ecosystem health. For example, many native defoliating insects help return nutrients to the soil and may provide an important food source (e.g. caterpillars) for migratory birds^35^. Moreover, forest disturbances tend to have a positive influence on indicators of biodiversity, including species richness and habitat quality^36^. A more practical consideration is that high total basal area values can arise under contrasting circumstances: A young plantation with high stem density and an old-growth tract with low stem density could both have high total basal area values, yet differing levels of vulnerability to insects and pathogens (as influenced by density but also tree age, size, species, and other factors). Still, the negative ecological and economic consequences of forest insect and pathogen disturbances are substantial and obvious, reemphasizing the need for tools that facilitate continued and improved monitoring and assessment.

## Results

**Figure 1:**
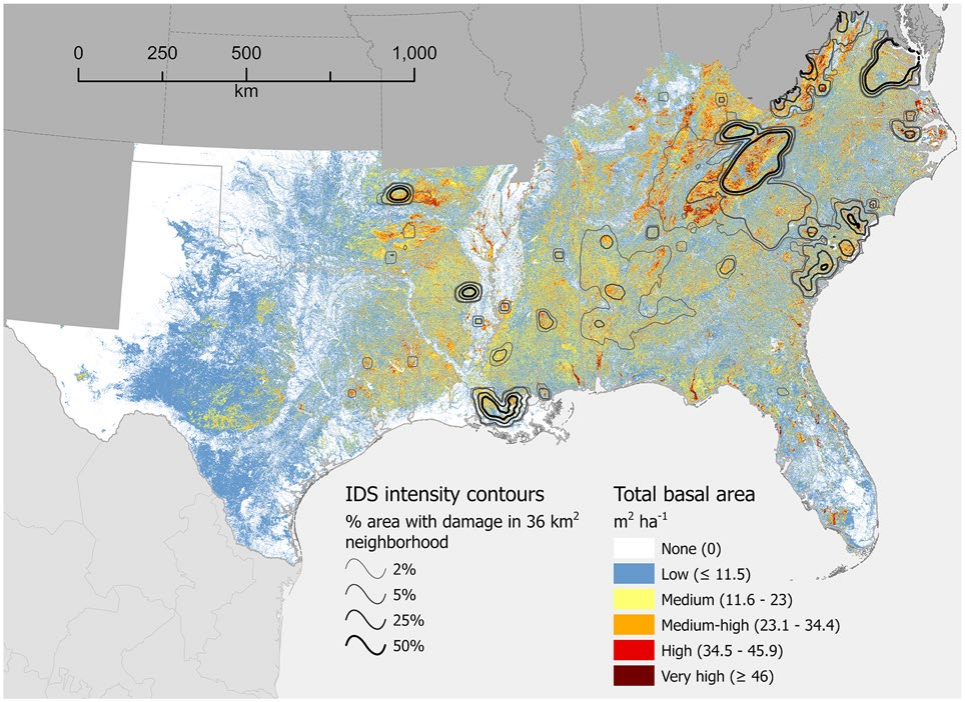
Total basal area (TBA), m^2^ ha^−1^, of forestland across the South region of the conterminous USA. Values represent averages across 240-m resolution map cells. Many cells contain areas of non-forest, which causes their values to be lower than TBA values typical of the constituent forest stands. Cells with a complete lack of tree cover are shown in white. Contour intervals depict relative concentrations of forest damage (defoliation or mortality) as recorded in Insect and Disease Survey (IDS) data. For example, the 50% contour line defines places where at least half of the area within a 36 km^2^ neighborhood experienced damage at least once during the analytical period, 2000–2019. See “Methods” for analytical details. Map created using Esri ArcGIS Pro 2.9.5, https://www.esri.com/en-us/arcgis/products/arcgis-pro/overview.

**Figure 2:**
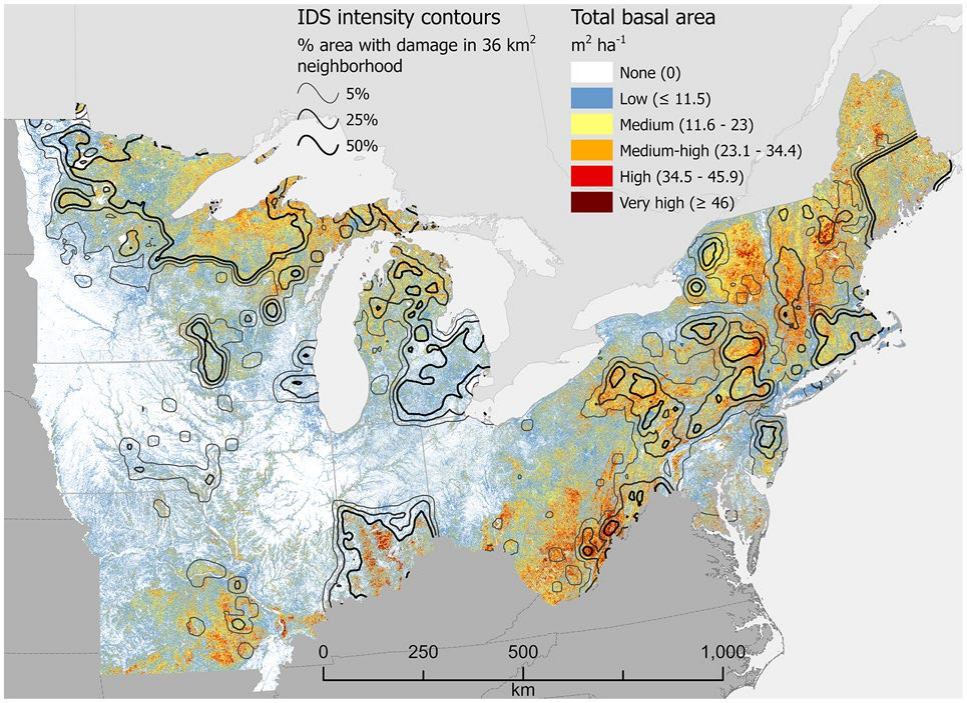
Total basal area (TBA), m^2^ ha^−1^, of forestland across the North region of the conterminous USA. Values represent averages across 240-m resolution map cells. Many cells contain areas of non-forest, which causes their values to be lower than TBA values typical of the constituent forest stands. Cells with a complete lack of tree cover are shown in white. Contour intervals depict relative concentrations of forest damage (defoliation or mortality) as recorded in Insect and Disease Survey (IDS) data. For example, the 50% contour line defines places where at least half of the area within a 36 km^2^ neighborhood experienced damage at least once during the analytical period, 2000–2019. See “Methods” for analytical details. Map created using Esri ArcGIS Pro 2.9.5, https://www.esri.com/en-us/arcgis/products/arcgis-pro/overview.

**Figure 3:**
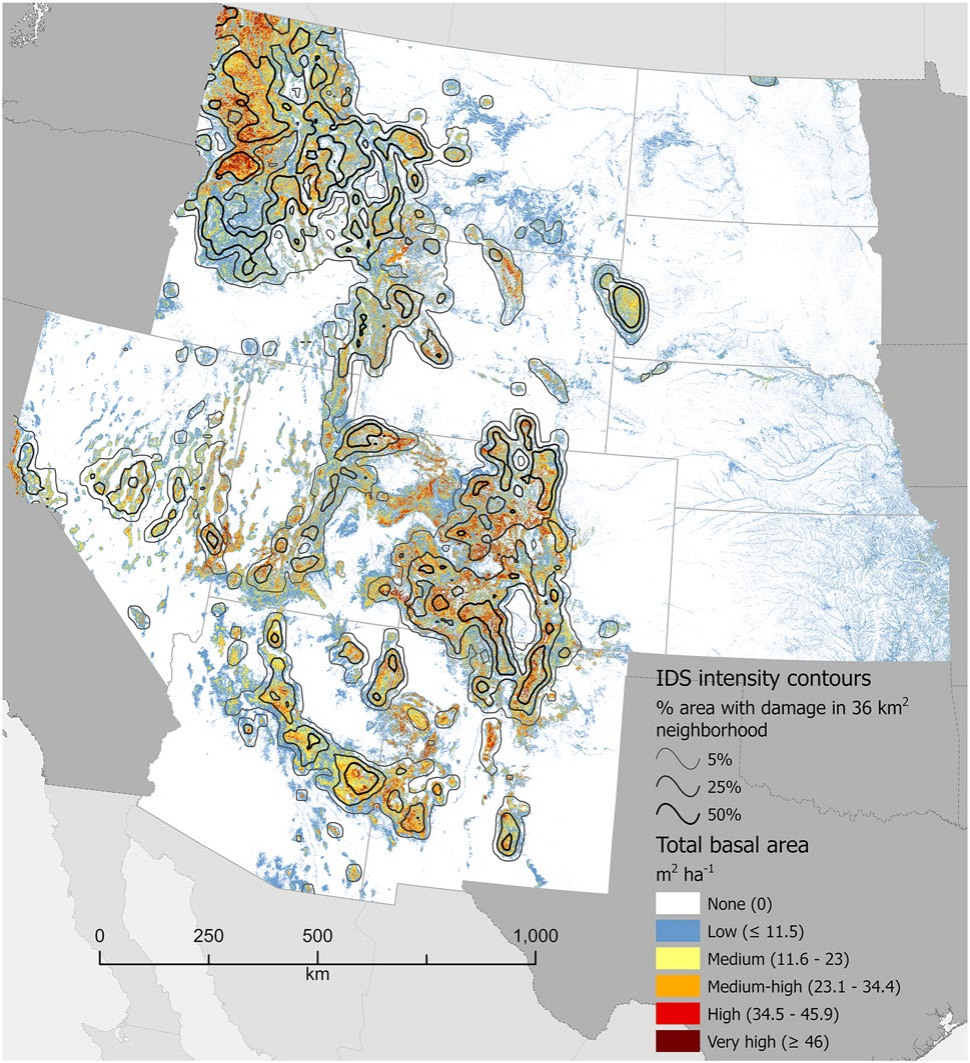
Total basal area (TBA), m^2^ ha^−1^, of forestland across the Interior West region of the conterminous USA. Values represent averages across 240-m resolution map cells. Many cells contain areas of non-forest, which causes their values to be lower than TBA values typical of the constituent forest stands. Cells with a complete lack of tree cover are shown in white. Contour intervals depict relative concentrations of forest damage (defoliation or mortality) as recorded in Insect and Disease Survey (IDS) data. For example, the 50% contour line defines places where at least half of the area within a 36 km^2^ neighborhood experienced damage at least once during the analytical period, 2000–2019. See “Methods” for analytical details. Map created using Esri ArcGIS Pro 2.9.5, https://www.esri.com/en-us/arcgis/products/arcgis-pro/overview.

**Figure 4:**
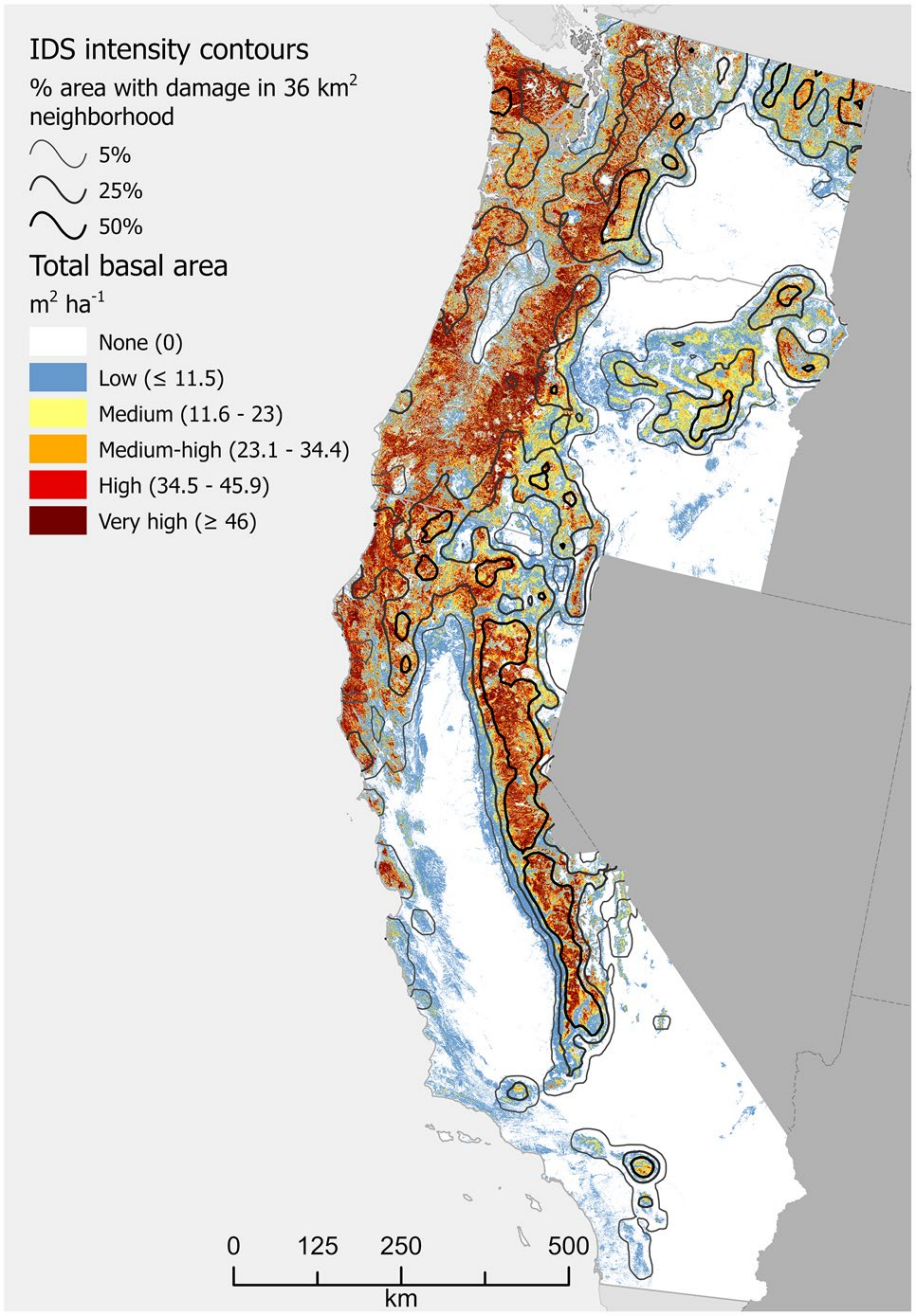
Total basal area (TBA), m^2^ ha^−1^, of forestland across the West Coast region of the conterminous USA. Values represent averages across 240-m resolution map cells. Many cells contain areas of non-forest, which causes their values to be lower than TBA values typical of the constituent forest stands. Cells with a complete lack of tree cover are shown in white. Contour intervals depict relative concentrations of forest damage (defoliation or mortality) as recorded in Insect and Disease Survey (IDS) data. For example, the 50% contour line defines places where at least half of the area within a 36 km^2^ neighborhood experienced damage at least once during the analytical period, 2000–2019. See “Methods” for analytical details. Map created using Esri ArcGIS Pro 2.9.5, https://www.esri.com/en-us/arcgis/products/arcgis-pro/overview.

**Figure 5:**
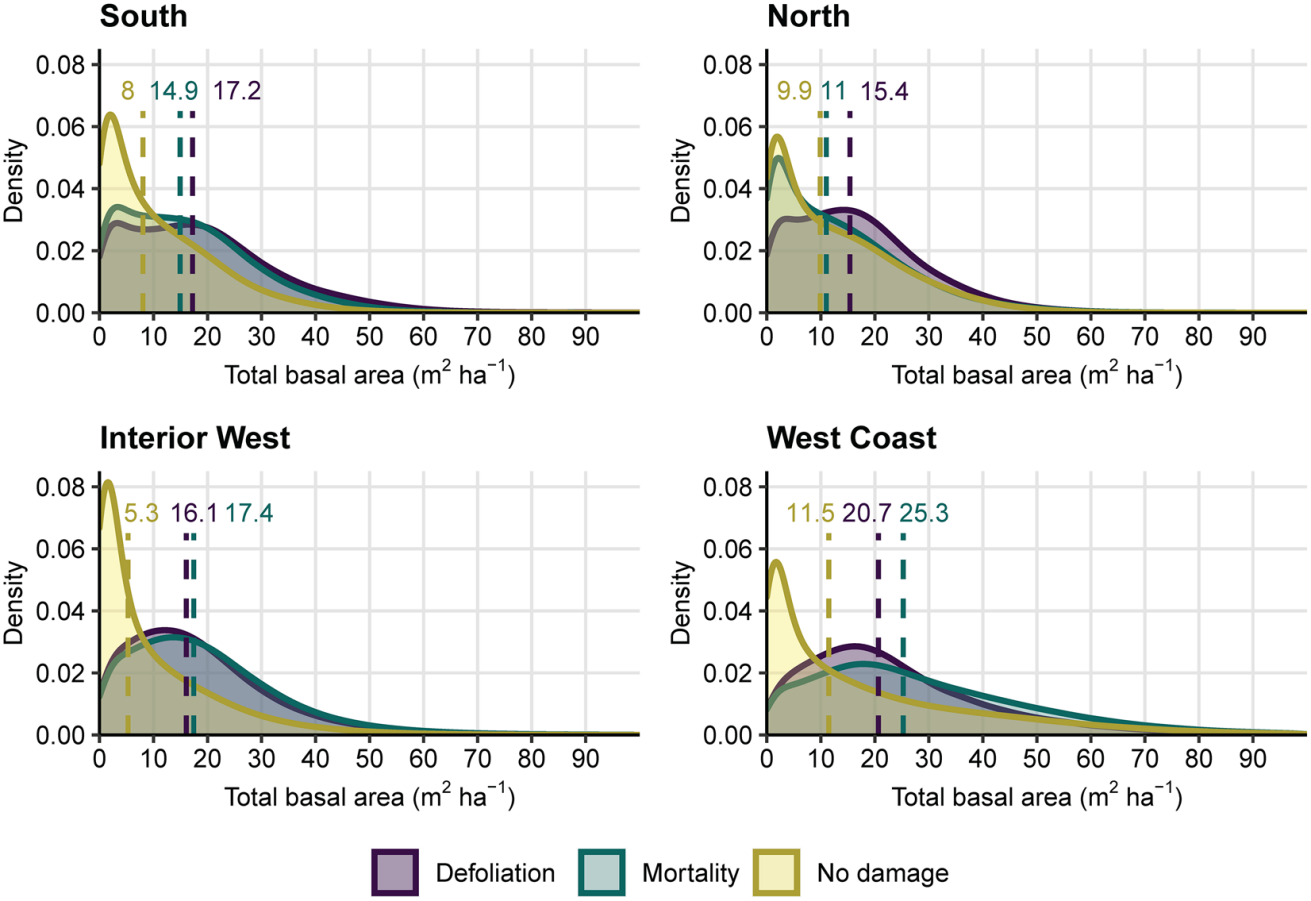
Probability density plots, by region. Shaded plots depict the distributions of total basal area (TBA) values for three groups of observations (map cells): Defoliation = all forested (i.e. TBA > 0) cells where defoliation was recorded at least once from Insect and Disease Survey (IDS) data in the analysis period, 2000–2019; Mortality = all forested cells where mortality was recorded at least once in the period; No damage = all forested cells where neither defoliation nor mortality was recorded during the period. Median TBA values for the three groups are indicated by vertical dotted lines, each of which is labeled in the corresponding color.

Total basal area (TBA), in five range categories from low (≤ 11.5 m^2^ ha^−1^) to very high (≥ 46 m^2^ ha^−1^), is shown for four regions of the conterminous USA: South, North, Interior West, and West Coast (Figs. 1, 2, 3 and 4, respectively). Overall, forests of the West Coast region (Fig. 4) are significantly denser than any other region. Overlaid on each regional TBA map in Figs. 1, 2, 3 and 4 is a set of contour lines depicting the relative spatial intensity of locations with insect- or pathogen-caused forest damage (defoliation and/or mortality) documented in Insect and Disease Survey (IDS) data during 2000–2019. Generally, forested areas exhibiting high damage intensity tended to have higher TBA values than nearby areas with lower damage intensity, although there were exceptions, most notably southeastern Michigan (Fig. 2). This tendency is reflected in basic summary statistics for the regions (Table 1): Other than in the North region, the median TBA of locations with defoliation or mortality was 2–3 times higher than the median TBA for locations where neither type of damage was recorded. Furthermore, in all four regions, pairwise comparisons (Dunn’s test) of the frequency distributions of TBA values among locations with defoliation, mortality, or no damage showed highly significant (*p* < 0.0001) differences between the three groups (Fig. 5, Table 2). In other words, map cells with defoliation or mortality generally had higher TBA values in all regions than cells without damage. Despite being statistically different, the TBA frequency distributions of the defoliation and mortality groups resemble each other in every region except the North, where the distribution for the mortality group is much more similar to the distribution for the no damage group. This is echoed in the *z* test statistics from the multiple comparisons (Table 2), where the North (smallest *z* = 89.06, for mortality – no damage) deviated from the pattern in the other three regions (i.e. smallest *z* for mortality – defoliation).

**Table 1:** Summary statistics for four geographic regions of the conterminous USA.

Region	*n* (# cells)	Area (M ha)	% Region	TBA			
				Med	MAD	Q1	Q3
South							
Defoliation	904,089	5.2	3.5	17.2	14.0	8.3	27.1
Mortality	217,905	1.3	0.9	14.9	12.6	6.9	24.1
No damage	24,374,181	140.4	95.7	8.0	9.5	2.5	17.0
North							
Defoliation	3,504,833	20.2	18.6	15.4	11.9	7.8	23.9
Mortality	1,507,104	8.7	8.0	11.0	11.9	3.7	20.4
No damage	14,118,192	81.3	74.8	9.9	11.6	3.0	20.0
Interior West							
Defoliation	1,500,054	8.6	11.0	16.1	11.9	8.7	25.5
Mortality	2,917,186	16.8	21.3	17.4	12.9	9.4	27.1
No damage	9,826,344	56.6	71.8	5.3	6.8	1.4	14.7
West Coast							
Defoliation	331,334	1.9	4.8	20.7	15.0	11.7	32.8
Mortality	1,895,254	10.9	27.6	25.3	19.1	14.0	41.1
No damage	4,777,310	27.5	69.5	11.5	15.0	2.8	29.2

Statistics are reported for three groups: Defoliation = all forested (i.e. total basal area > 0) map cells where defoliation was recorded at least once from Insect and Disease Survey (IDS) data in the analysis period, 2000–2019; Mortality = all forested cells where mortality was recorded at least once in the period; No damage = all forested cells where neither defoliation nor mortality was recorded during the period. Area (in M ha) is the combined area of all cells in the group and is also reported as a percentage of all forested cells in the region. For total basal area (TBA) values: *Med* median; *MAD* mean absolute deviation; *Q1* 1st quartile; *Q3* 3rd quartile.

**Table 2:** Dunn’s test results for four geographic regions of the conterminous USA.

Comparison	Region							
	South		North		Interior West		West Coast	
	*z*	*p*	*z*	*p*	*z*	*p*	*z*	*p*
Defoliation—mortality	57.82	< 0.0001	327.73	< 0.0001	− 59.41	< 0.0001	− 95.86	< 0.0001
Defoliation—no damage	603.28	< 0.0001	662.82	< 0.0001	904.55	< 0.0001	242.08	< 0.0001
Mortality—no damage	236.15	< 0.0001	89.06	< 0.0001	1278.74	< 0.0001	716.86	< 0.0001

Multiple pairwise comparisons of the distributions of total basal area (TBA) values between three groups: Defoliation = all forested (i.e. TBA > 0) map cells where defoliation was recorded at least once from Insect and Disease Survey (IDS) data in the analysis period, 2000–2019; Mortality = all forested cells where mortality was recorded at least once in the period; No damage = all forested cells where neither defoliation nor mortality was recorded during the period. Table 1 shows the number of observations (map cells) *n* in each group. Individual *z* test statistics shown for each pairwise comparison; *p*-values corrected using the Benjamini–Hochberg adjustment.

Table 3 lists the most common damage-causing agents across the four regions based on cumulative IDS data. In both western regions, the western spruce budworm (*Choristoneura freemani*), a defoliator, and native bark beetles with conifer hosts – pine, spruce (*Picea* spp.), fir (*Abies* spp.), and Douglas-fir (*Pseudotsuga menziesii*) – were dominant. Bark beetles are recognized globally as major forest disturbance agents that can cause extensive tree mortality during outbreaks^37^. Bark beetle populations have expanded considerably across the western USA over the last few decades in concert with drought and warming temperatures, conditions that lead to tree stress and provide opportunities for these insects to expand their elevational and latitudinal ranges as well as their activity periods^38,39^. Native bark beetles (i.e. southern pine beetle and engraver beetles, *Ips* spp.) were also fairly prominent in the South, along with several defoliators that typically prefer pine or oak hosts. Their prevalence reflects the relative abundance of conifer-dominated forest types in the West and pine- or oak-dominated forest types in the South. In the North, a more distinctive set of agents is important, which is consistent with a greater variety of forest types dominated by hardwoods other than oaks (e.g. maple, *Acer* spp.; birch, *Betula* spp.) and the abundance of spruce and eastern hemlock (*Tsuga canadensis*) in the more northerly portions of this region. Furthermore, pine-dominated forests, and thus pine bark beetles, are less abundant in the North. However, what is perhaps most distinctive about the North is that three of its most common damage agents are invasive alien insect species: emerald ash borer (*Agrilus planipennis*), spongy moth, and hemlock woolly adelgid *(Adelges tsugae)* (Table 3). This is because of the relative abundance of their hosts in the North, but also because they were first introduced to this region and have been present there the longest^40^.

**Table 3:** The most common damage-causing agents (insects, pathogens, declines, or complexes) in four geographic regions of the conterminous USA.

South	North	Interior West	West Coast
Fall cankerworm** *Alsophila pometaria*	Spongy moth** *Lymantria dispar dispar*	Western spruce budworm** *Choristoneura occidentalis*	Fir engraver* *Scolytus ventralis*
Yellow poplar weevil** *Odontopus calceatus*	**Emerald ash borer *Agrilus planipennis***	Mountain pine beetle* *Dendroctonus ponderosae*	Mountain pine beetle* *Dendroctonus ponderosae*
Forest tent caterpillar** *Malacosoma americana*	Forest tent caterpillar** *Malacosoma americana*	Spruce beetle* *Dendroctonus rufipennis*	Western pine beetle* *Dendroctonus brevicomis*
Baldcypress leafroller** *Archips goyerana*	Spruce budworm** Choristoneura fumiferana	Pinyon needle scale *Matsucoccus acalyptus*	Western spruce budworm** *Choristoneura occidentalis*
Jumping oak gall wasp** *Neuroterus* sp.	Eastern larch beetle* *Dendroctonus simplex*	{Subalpine fir mortality complex}	Jeffrey pine beetle* *Dendroctonus jeffreyi*
Loblolly pine sawfly** Neodiprion taedae lineari	Bronze birch borer *Agrilus anxius*	Douglas fir beetle* *Dendroctonus pseudotsugae*	[Swiss needle cast] *Phaeocryptopus gaeumannii*
Spongy moth** *Lymantria dispar dispar*	Yellow poplar weevil** *Odontopus calceatus*	{Five-needle pine decline}	Douglas fir beetle* *Dendroctonus pseudotsugae*
Ips engraver beetles* *Ips* spp.	**Hemlock woolly adelgid *Adelges tsugae***	Fir engraver *Scolytus ventralis*	Pine butterfly** *Neophasia menapia*
Southern pine beetle* *Dendroctonus frontalis*	Fall cankerworm** *Alsophila pometaria*	{Sudden aspen decline}	Flatheaded fir borer *Melanophila drummondi*
**Emerald ash borer *Agrilus plannipennis***	Winter moth** *Operophtera brumata*	Ips engraver beetles* *Ips* spp.	**[Sudden oak death] *Phytophthora ramorum***

Regional ranks, from top to bottom, are based on the cumulative number of hectares with damage (defoliation or mortality) in Insect and Disease Survey (IDS) data from 2009 to 2019. Conifer bark beetles are indicated with an asterisk (*) to highlight the importance of this group, particularly in the western USA where there is a much greater diversity of bark beetle species with tree-killing potential. Insect defoliators are shown by two asterisks (**). Non-native invasive species are highlighted in bold type. Brackets [ ] denote a pathogen. Braces { } denote a complex or decline, where multiple biotic and abiotic factors interact to diminish tree health in ways that are poorly understood.

The emerald ash borer is especially noteworthy as the primary reason that southeastern Michigan departed from the typical pattern observed across the regions of high damage intensity coinciding spatially with high TBA values (Fig. 2). This part of Michigan is where the emerald ash borer was first recorded in the USA and was an early epicenter of ash (*Fraxinus* spp.) decline and mortality^41^. Despite the insect’s obvious impacts, a study conducted in this locale found no relationship between ash mortality caused by the insect and forest attributes including tree species diversity, the relative dominance of ash, or TBA^42^.

Notwithstanding such exceptions, odds ratios from logistic regression models for the regions (Supplementary Table S1) show a consistent positive association between TBA and the likelihood of damage (mortality or defoliation) from insects and pathogens. A 1 m^2^ ha^−1^ increase in TBA increased the odds of damage occurrence by ≈5% in the South and Interior West regions and by ≈2% in the North and West Coast regions (see Supplementary Table S1). These results demonstrate that, at a regional scale, TBA is a significant indicator of pest disturbance regardless of forest composition and the mix of native and non-native damage agents involved.

## Discussion

The South and Interior West have higher odds ratios between TBA and pest damage likelihood because the most relevant hosts (e.g. pines and oaks in the South) comprise a very large share of the TBA in many forest stands, meaning that TBA and host tree BA are correlated with respect to the regions’ most important damage agents (Table 3). The lower odds ratio for the West Coast region may be explained by a higher baseline for TBA relative to the other regions, such that a one-unit increase in TBA causes less of a change in the odds of damage occurrence. In contrast, the comparatively lower odds ratio in the North region likely stems from the aforementioned prominence of invasive alien species among the region’s main forest damage agents, both recently (Table 3) and historically^40^.

Some elaboration is needed about the impacts of native versus non-native insects and pathogens in relation to basal area. The damage depicted in the IDS data is largely caused by native forest insects and pathogens (Table 3), despite the increasing number of consequential non-native species that continue to become established in the USA^43^. In comparison to native species, many of the most destructive non-native species attack hosts that are less abundant on the landscape or that are primarily found in the understory, or are diseases that kill very slowly over many years (e.g. white pine blister rust caused by the fungus *Cronartium ribicola*)^44^. Thus, the damage resulting from these agents may not be easily discerned from aircraft or from satellite imagery because their damage footprints are smaller or more diffuse.

Furthermore, because of long-standing coevolutionary forces, native insects and pathogens generally do not reach damaging levels unless their host trees are subjected to enough environmental stress to compromise their physical and chemical defense systems (conifer resin, terpenes, tannins, etc.)^29,35,45,46^. Tree stress occurs for a variety of reasons – indeed, overall stress from biotic and abiotic causes is expected to increase in most forests under a warming climate^3,47^ – but can be exacerbated in dense stands where trees compete more vigorously with one another for available light, water, and nutrients^12,29^. On the other hand, some non-native species, as perhaps best exemplified by the emerald ash borer^41,42^, will kill their hosts regardless of health status or density in invaded sites. This is mostly thought to be due to a combination of the host’s lack of coevolutionary resistance to the invader and the ability of the latter to thrive in a new habitat in the absence of its cohort of natural enemies, which are other insects and pathogens that are adapted to attack or compete with the invader in its native habitat and that keep its populations in check^48,49^. The ramifications of high TBA are therefore ambiguous with respect to non-native insects and pathogens. In a high-TBA forest stand, the risk of outbreaks is presumably increased if a large proportion of the trees are suitable hosts for at least one non-native insect or pathogen of concern, in keeping with the resource concentration hypothesis^27,50,51^. However, if most of the trees in the stand are not hosts, then the risk is probably decreased, based on the hypothesis of resource dilution^29,50^. Across larger regions, forests are likely to experience both resource concentration and dilution effects depending on the suite of pests to which they are exposed locally in conjunction with host availability, which is only partially moderated by overall density (i.e. TBA). Conceivably, in a region where a large share of the forest insects and pathogens are non-native invaders, TBA would have limited association with disturbance potential. In our analyses, the North region came closest to this scenario, but a majority of the region’s important damage-causing agents (Table 3) were native despite the presence of several highly damaging non-native insects and diseases.

Another complication is that high TBA values can occur under differing circumstances, such as the example given earlier of a young plantation with tight spacing between trees versus an old-growth stand with comparatively fewer but much larger trees. The distinction in this example may be particularly apt, based on evidence that mature and old-growth forests can be highly resilient to disturbance^28,52^. Furthermore, tree species that one might naturally associate with high-TBA, old-growth conditions, such as the giant sequoia (*Sequoiadendron giganteum*), have demonstrated – through their longevity – resistance to insects, pathogens, and other disturbance agents^53^. On the other hand, high-TBA (> 27.6 m^2^ ha^−1^) forests are relatively common across age classes throughout the USA, and in the South region they comprise a large percentage of young (≤ 40 years) forests (see Supplementary Fig. S1). Thus, there are abundant forests for which TBA remains a pertinent metric with respect to insect and pathogen outbreaks.

Other metrics related to the density, size distribution, or configuration of trees in a stand may also be meaningful at regional or broader spatial scales, such as Reineke’s stand density index (SDI) or the Hegyi competition index^4,22,23^. An advantage of TBA is that it is computationally straightforward and therefore easily understood by users, including those unfamiliar with forest mensuration and inventory^54^. By comparison, SDI incorporates information about site quality and tree species, thus perhaps providing a more robust metric of relative density, but such information is seldom available for every forest location at regional scales. TBA, however, can be calculated from field measurements that are standard for any national or regional forest inventory, though our case study was limited to the conterminous USA. Because TBA requires data about tree diameters, it may not be possible to calculate and map TBA globally, as has been done for tree density^11^. Nevertheless, we believe its data requirements are modest enough that it should have wide geographic applicability as an indicator.

### Potential broad-scale applications of TBA

Given its constraints, what are appropriate uses of TBA as an indicator? Recall that the scope of our analyses was limited to insect and pathogen disturbances. While we demonstrated that TBA is relevant to this dimension of forest health at broad scales, we acknowledge that it provides incomplete information about relationships and dynamics – between agents, hosts, environment, etc. – that can operate in a complex manner at multiple scales. Hence, we caution against interpreting our results as prescriptive of forest management (or of a decision not to manage). Indeed, while much research supports the use of silvicultural treatments to create pest-resistant forests, the effective mechanisms remain unclear^29^. Furthermore, because management responses are usually reactive to pest outbreaks rather than proactive^27^, they can lead to more pest-prone forests^29^. At the stand level, thinning or other silvicultural treatments to reduce basal area have shown mixed results with respect to decreasing insect and pathogen risk^55–57^ , and even when effective, may not prevent major outbreaks because of the impracticality of implementing treatments over large areas, especially when there are overriding effects of climatic stress (e.g. from hot droughts)^58,59^.

Nonetheless, it is worth revisiting what prompted us to investigate the utility of TBA as a metric. Most areas of the USA are growing more wood volume than they harvest^60^, leading to overly dense forests in some places that are more susceptible to drought and temperature stress associated with a changing climate^9,20,23,39^. Chronic and worsening bark beetle outbreaks are a poignant illustration of this, particularly in conifer forests of the western USA^9,39^, while hardwood forests in the eastern USA are increasingly composed of aging tree cohorts that are under threat of eventual replacement by non-native, invasive plants and native tree species that support less biodiversity^60^. Although research points to greater tree species diversity at the stand level as an effective way to mitigate insect and pathogen disturbances in the face of climate change, for the sake of practicality, managers also have to consider landscape- and regional-scale factors that influence adaptation efforts^27^. We propose that TBA, when summarized at a regional or national scale (e.g. represented with a raster map as in our analyses), can serve as a useful first filter for identifying landscapes, forest types, or ecosystems that may be at risk of impacts from insect and pathogen disturbances, and therefore merit follow-up analysis – and possible mitigation actions – at a finer scale. This may be viewed as applying summarized TBA for strategic decision-making (i.e. which locations are the highest priority for managers) rather than tactical decision-making (i.e. which management actions are appropriate for specific locations given their biophysical characteristics) in forested areas^61^.

For TBA to be most useful in these circumstances, it should be based on the most current inventory data that are available. It may be particularly valuable to track change in TBA over time when and where the appropriate data exist. Landscape- or regional-level increases in TBA over a decade or more, for example, may imply increasing broad-scale susceptibility to native insect or pathogen disturbance, while stable or decreasing TBA could indicate lower susceptibility. The implications of TBA change for insect and pathogen disturbance will, of course, depend on forest composition and stand age, among other factors; all of these would need to be accounted for in assessing the potential repercussions of TBA change. Whether considering TBA at a single point in time or TBA change over time, further analyses of the relationships between forest stand age and composition on one hand and TBA-associated pest disturbance risk on the other could help to identify more precisely those forest areas that are most in need of insect and pathogen disturbance monitoring and mitigation.

## Methods

### Data sources

We used two USDA Forest Service data sets for our analyses: Insect and Disease Survey (IDS) data compiled by the national Forest Health Monitoring (FHM) Program and a map of total basal area (TBA) developed principally from field plot data collected and administered by the Forest Inventory and Analysis (FIA) Program. Through the IDS, the FHM Program (https://www.fs.usda.gov/foresthealth/protecting-forest/forest-health-monitoring/) annually documents major forest disturbances, both biotic and abiotic, across the United States. Key information collected for each disturbance includes the disturbance agent, type of damage (mortality, defoliation, branch breakage, crown discoloration, etc.), the primary tree host(s) affected, and timing of the disturbance^62^. Agents documented in the IDS data are predominantly insects or fungi, but can include other organisms (e.g. animals, bacteria) or abiotic disturbances usually associated with weather (frost, hail, wind, flooding, drought, etc.). Over the last 20 years, the top damage-causing agents by cumulative area impacted are mostly native insect species, the majority of which are conifer-killing bark beetles or moth caterpillar defoliators (Table 3). The IDS data are geospatial in nature, with disturbances depicted by polygon or point features. The data are collected primarily through aerial survey of forestland in small aircraft by highly trained forest health specialists, although remotely sensed imagery from satellites is increasingly being used to monitor and map forest health conditions^63–65^. In either case, some ground survey for verification of the damage-causing agent by a specialist is necessary^66^. The IDS data have recognized limitations, perhaps most notably that they are collected in a targeted fashion rather than through a systematic, comprehensive survey effort. Furthermore, data attributes such as damage severity can be misestimated^67^, and some types of damage agents (e.g. disease-causing fungi such as root rots and stem cankers as well as agents infesting widely dispersed hosts) are virtually impossible to detect remotely. Thus, the IDS data are not fully comprehensive for all potential biotic disturbances. Nevertheless, they have served as a critical source of spatially explicit information on major forest disturbances in the USA since the late 1990s, although spatial databases for some insects or pathogens are decades older^13,15,32^ and aerial surveys for forest health purposes date back to the 1940s^66^. Several analyses involving IDS data have appeared in recent refereed literature^67–71^, emphasizing their potential utility. The IDS data also served as a key input to the USDA Forest Service’s Terrestrial Conditions Assessment (TCA), which was intended to identify restoration opportunities on national forest system (NFS) lands^61^.

The FIA Program (https://www.fia.fs.usda.gov/) focuses on measuring and monitoring changes in forest vegetation across a nationwide network of field inventory plots laid out systematically (approx. 1 plot per 2428 ha of forest) and based on a spatially balanced sampling design for conducting broad-scale statistical analyses of forest conditions^72^. Although some forest health data are collected on FIA plots (e.g. occurrence of damage-causing agents), these data are collected inconsistently and can be difficult to interpret^73,74^. Some of the difficulty arises from the panelized nature of the FIA sampling scheme: 20% or fewer of the plots are visited annually within each state (i.e. during one “panel”), with full coverage on 5- to 7-year cycles in the eastern USA and 10-year cycles in the western USA^72,75^. Compounded by the relatively low sampling intensity of the FIA design, plot visits may miss part or all of a disturbance event, spatially and/or temporally. Furthermore, FIA field crews have some training in diagnosing damage-causing agents but are rarely specialists in subject areas like entomology or pathology, so their agent identifications are not always reliable^73^. Nevertheless, FIA data are considered a definitive source of standardized and unbiased information about forest metrics such as tree density and basal area (BA)^76^. To deal with the sampling intensity limitation of the FIA data, a variety of model-based methods have been used to develop spatially continuous maps of these and other metrics, typically by combining FIA plot measurements with ancillary geospatial data, including satellite imagery as well as meteorological, topographic and other environmental variables^76–79^.

To depict total basal area (TBA) in a spatially continuous fashion, we used a 240-m resolution raster map^80^ that was developed from FIA plot data to support the 2013–2027 National Insect and Disease Risk Map (NIDRM) assessment^81,82^. Most of the FIA plots used as input were measured between 1999 and 2005, although some were measured as late as 2009. Consequently, a large majority of the plot measurements predate the damage observations recorded in the IDS data. Tree data extracted from the plots were limited to live trees ≥ 2.54 cm DBH (i.e. sapling size or larger).

TBA was modeled from the FIA data using a regression tree approach implemented in Cubist version 2.07^83^. The Cubist software package utilizes piece-wise non-overlapping regression, where separate multivariate linear models are constructed for each rule in the output collection of rules comprising the final predictive model^83^. Independent variables (i.e. geospatial predictor layers) used in the modeling were derived from four different USA data sets generated at a 30-m resolution: Landsat imagery used to develop the National Land Cover Database (NLCD); USDA Natural Resource Conservation Service (NRCS) localized and regionalized soils data; the National Elevation Dataset; and standard climate normal data (e.g. annual precipitation, mean temperature in the warmest month) spatially modeled from nearly 8000 weather stations across the country for the period 1971–2000. A full list of predictors used in the broader NIDRM assessment is provided in Appendix A of Ellenwood et al.^81^, although only some of these predictors were used to model TBA.

Additional details about the modeling approach are provided in Krist et al.^82^. The Cubist model output was converted to a raster data set with 30-m resolution, which was then resampled to a 240-m data set for national- and regional-scale application. Note that the TBA values in this subsequent data set represent averages across the 5.76-ha area of each cell, and many individual cells likely include sub-areas of non-forest that reduce these averages. Thus, the reported TBA values may underrepresent actual TBA values of the constituent forest stands.

### Data processing

We divided the conterminous USA (i.e. excluding Alaska and Hawaii) into four geographic regions (see Figs. 1, 2, 3 and 4): South (13 states), North (20 states), Interior West (12 states), and West Coast (3 states). All data processing and analyses were performed separately for these regions.

We used all available IDS geospatial data for the period 2000–2019^62^. For each year in the 20-year analytical period, we selected all IDS features (points or polygons) that recorded defoliation or mortality caused by a relevant biotic agent: insect, disease, animal, or multi-agent complex. Across the data set, the vast majority of damage occurrences were caused by insects or pathogens (primarily fungi). We omitted features that documented instances of minor damage (e.g. discoloration, branch breakage). Next, we converted the selected features to raster format using the same spatial reference and resolution (240 m) as the TBA layer described below. Any point feature or polygon feature less than 5.76 ha in size (the area of a 240-m cell) was converted to a single corresponding map cell. The result was a set of two binary raster maps for each year, one for mortality and another for defoliation, where a cell value of 1 indicated the occurrence of the associated damage type caused by at least one agent (0 otherwise). Using map algebra, we combined each stack of 20 annual raster maps into a single binary raster map summarizing the analytical period, where a cell value of 1 indicated the occurrence of mortality (or defoliation) during at least one year in the 20-year period (0 otherwise).

### Statistical analysis

For analytical purposes, we treated each map cell location, with its corresponding values for the summary mortality and defoliation maps as well as the TBA raster map, as an individual observation. We assembled three groups of observations for each region: “Mortality”, comprised of observations (cells) where mortality occurred at least once during the analytical period; “Defoliation”, comprised of observations where defoliation occurred at least once during the period; and “No damage”, comprised of observations where neither mortality nor defoliation was recorded during the period. We limited the groups to forested observations only (i.e. cells where TBA > 0) and excluded all others from analysis. We included observations where both mortality and defoliation were recorded during the analytical period in each of these groups. Overlap between the mortality and defoliation groups was relatively limited, ranging from 0.1 (South) to 4.1% (Interior West) of a region’s forested observations. For each region, we computed Dunn’s test for multiple pairwise comparisons of the TBA value distributions of the three groups using rank sums; all *p*-values were corrected using the Benjamini–Hochberg adjustment.

We constructed logistic regression models for each region, with damage (= 1)/no damage (= 0) as the binary outcome variable and TBA as the sole predictor. We assigned observations (forested cells) with recorded mortality and/or defoliation to the “damage” category – thus ignoring any overlap – and assigned all other observations to the “no damage” category. We computed odds ratios as a measure of association between TBA and the likelihood of damage. Since prediction was not one of our objectives, we used each region’s full data set to construct the models; partitioning the data randomly into 80% training and 20% test sets resulted in negligible changes to the model coefficient estimates and odds ratios. We performed all statistical tests and modeling procedures in the R version 4.1.1^84^ software environment.

To illustrate possible associations between the IDS and TBA data sets, we created a single binary raster map (damage/no damage) for each region. Consistent with the input for the logistic regression models, we assigned map cells with recorded mortality and/or defoliation to the “damage” category and all other cells to the “no damage” category. We applied the *Aggregate* function in ArcGIS Pro version 2.8.0^85^ with an aggregation factor of 25 to develop a new 6-km resolution raster map for visualization purposes. Cell values in this new map were scaled from 0 to 100, representing the percentage of each 6-km cell’s area (i.e. the percent share of the 625 underlying 240-m cells) that were in the damage category in the original map. We applied a 5 × 5 focal mean filter to smooth this map prior to developing a set of contour intervals from the percentage values.

## Supplementary Information

Supplementary Information.

## Data Availability

All intermediate data sets generated and/or analyzed for this study are available from the corresponding author upon reasonable request. Furthermore, all primary data used in this study are publicly available from the US Department of Agriculture, Forest Service, Forest Health Protection: https://www.fs.usda.gov/foresthealth/applied-sciences/mapping-reporting/detection-surveys.shtml; https://www.fs.usda.gov/foresthealth/applied-sciences/mapping-reporting/indiv-tree-parameter-maps.shtml.
