# Supplementary material for: Denser forests across the USA experience more damage from insects and pathogens
Source: Sci Rep. 2023 Mar 4;13:3666. doi: 10.1038/s41598-023-30675-z (PMC9985637; doi:10.1038/s41598-023-30675-z)

Supplementary Information

Denser forests across the USA experience more damage from insects and pathogens

Christopher Asaro, Frank H. Koch, and Kevin M. Potter

Supplementary Table S1. Odds ratios for each region from logistic regression models with damage/no damage as the binary outcome and total basal area (TBA) as a single predictor. Damage (=1) includes all observations (map cells) where defoliation or mortality was recorded at least once from Insect and Disease Survey (IDS) data during the analysis period, 2000-2019. Models were constructed using the full data set (i.e., all forested map cells) for each region.

Region	*n* (% Damage (=1))	Odds Ratio (95% CI)	*p*
South	25,468,986 (4.3%)	1.04719 (1.04705 – 1.04733)	<0.0001
North	18,887,015 (25.2%)	1.01940 (1.01931 – 1.01948)	<0.0001
Interior West	13,686,755 (28.2%)	1.05399 (1.05389 – 1.05409)	<0.0001
West Coast	6,870,043 (30.5%)	1.01939 (1.01932 – 1.01947)	<0.0001

Supplementary Figure S1. Percentage of all live trees in each region by stand age class and basal area class. Summaries generated using the EVALIDator 2.0.5 application (https://apps.fs.usda.gov/fiadb-api/evalidator), which retrieves data from the USDA Forest Service, Forest Inventory and Analysis (FIA) database (FIADB). Summaries are based on the most recent data available for each state in the conterminous USA and are for forest land only. "Unavail." = stand age class unknown or unspecified.

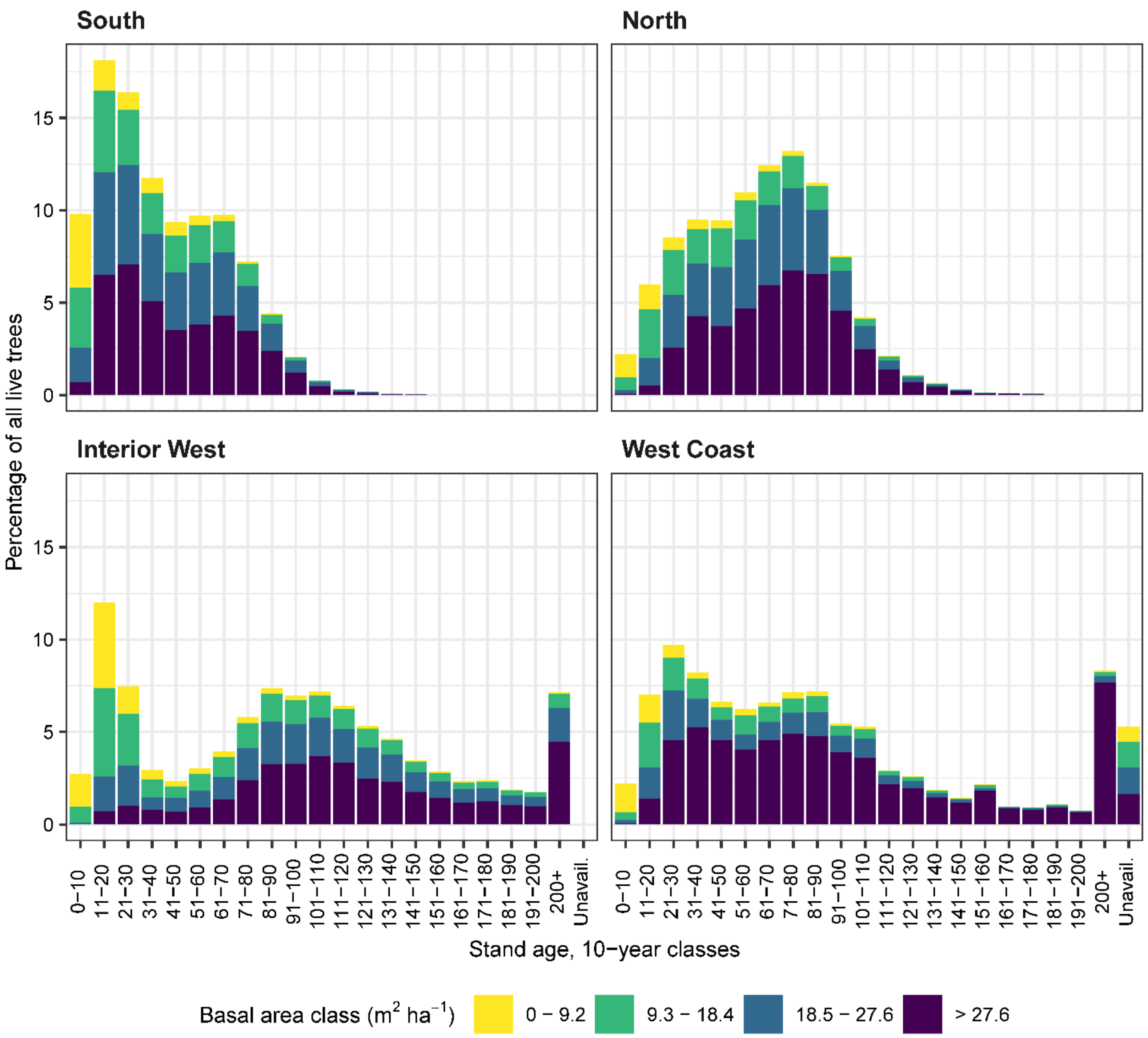

Supplement: Supplementary file 1 — Supplementary Information. [file 41598_2023_30675_MOESM1_ESM.pdf]
